# Investigation on the Sampling Frequency and Channel Number for Force Myography Based Hand Gesture Recognition

**DOI:** 10.3390/s21113872

**Published:** 2021-06-03

**Authors:** Guangtai Lei, Shenyilang Zhang, Yinfeng Fang, Yuxi Wang, Xuguang Zhang

**Affiliations:** College of Communication Engineering, Hangzhou Dianzi University, Hangzhou 310018, China; guangtailei@hdu.edu.cn (G.L.); zsyl@hdu.edu.cn (S.Z.); yxwang@hdu.edu.cn (Y.W.); zhangxg@hdu.edu.cn (X.Z.)

**Keywords:** force myography, sampling frequency, channel number, gesture recognition

## Abstract

Force myography (FMG) is a method that uses pressure sensors to measure muscle contraction indirectly. Compared with the conventional approach utilizing myoelectric signals in hand gesture recognition, it is a valuable substitute. To achieve the aim of gesture recognition at minimum cost, it is necessary to study the minimum sampling frequency and the minimal number of channels. For purpose of investigating the effect of sampling frequency and the number of channels on the accuracy of gesture recognition, a hardware system that has 16 channels has been designed for capturing forearm FMG signals with a maximum sampling frequency of 1 kHz. Using this acquisition equipment, a force myography database containing 10 subjects’ data has been created. In this paper, gesture accuracies under different sampling frequencies and channel’s number are obtained. Under 1 kHz sampling rate and 16 channels, four of five tested classifiers reach an accuracy up to about 99%. Other experimental results indicate that: (1) the sampling frequency of the FMG signal can be as low as 5 Hz for the recognition of static movements; (2) the reduction of channel number has a large impact on the accuracy, and the suggested channel number for gesture recognition is eight; and (3) the distribution of the sensors on the forearm would affect the recognition accuracy, and it is possible to improve the accuracy via optimizing the sensor position.

## 1. Introduction

People use their fingers and hand joints to perform activities of daily life (ADL), like hand gestures. In addition, hands are also an effective means of expressing information. Sign language is a typical application of this. The contraction of the forearm muscles drives the movement of the finger joints. Therefore, measuring the contraction of muscle is an effective approach of identifying hand movements, such as electromyographic signals and force myoelectric signals. Surface EMG is the most commonly used signal for measuring subcutaneous muscle activities, which has been applied in human–computer interaction. However, the electrodes for gathering EMG signals are susceptible to external disturbances and the signal quality is vulnerable to electromagnetic interference [1]. Besides, surface electromyography (sEMG) can hardly reflect changes happening in underlying muscles correctly [2]. Literature has shown that the FMG signal is the best replacement for EMG signals [3]. Its superiorities are mainly reflected in the following aspects: (1) the force morphology signal is insensitive to external interference; (2) the cost of the sensor and its conditioning circuit is lower; (3) muscle fatigue has a negligible effect on the FMG signal [4]; and (4) the force morphology signal outperforms the EMG signal in the same gesture recognition (84.6% for EMG vs. 96.7% for FMG [5]). The history of force morphology exploration can be traced back to the 1860s [6]. However, it was not until the late 1990s that researchers began to explore the force morphology signal to control prosthetic hands [7]. Related studies have shown that the amplitude of force morphology is linearly related to grip force [8]. In this paper, we focus on the effect of sampling frequency and the number of channels of FMG signal on the accuracy of gesture recognition.

There is no consistent standard of sampling frequency for FMG signal capturing, as can be seen in Table 1. The highest sampling frequency reaches up to 1 kHz, while the lowest sampling frequency is only 10 Hz. Li et al. [9] used 32 FSR sensors to collect signals (100 Hz) for seventeen actions and obtained an accuracy of 99%. Ha et al. [10] simultaneously recorded FMG signal (25 Hz) and hand gesture signals by a virtual motion glove and demonstrated plausible classification accuracy by SVM classification. Xiao et al. [11] applied FMG in virtual reality and obtained an accuracy of 93.4% for classifying six movements, where the FMG signals are sampled at frequency of 50 Hz. Carlo et al. [12] collected the data from six healthy volunteers’ while performing six actions at 1 kHz sampling frequency and obtained an accuracy rate of 92.33% by using a non-kernel ELM test with s-type functions.

Theoretically, the sampling frequency for FMG signal capturing is determined by the following two factors: First, the responding speed while pressuring the piezoelectric material. It only makes sense when the responding speed is fast enough for selecting higher sampling frequency. Second, the financial cost and power consumption. Higher sampling frequencies and more sensing channels would require higher performance of hardware (e.g., Analog-to-digital converter), which both increase the financial cost and power consumption [25]. However, it is obvious that using higher sampling resolution in both the temporal and spatial domain can improve the hand gesture recognition accuracy. Therefore, it is worthy to investigate how to balance the accuracy and cost, through reducing the sampling frequency and channel number.

Few studies have investigated the minimum sampling frequency for dynamic actions of forearm FMG signals. Carlo et al. [26] collected the data from twelve non-disabled volunteers and studied the effect of signal sampling frequency on the integrity of the force morphology signal. In this experiment, the twelve volunteers were asked to repeat hand gestures as fast as possible. After analyzing the results, it was suggested that the minimum sampling frequencies are 54 Hz and 58 Hz for sensing forearm and wrist FMG signals, respectively, during isometric movements, and 70 Hz and 84 Hz are needed during dynamic movements. Upper limb movements involve the joints of forearm, wrist and fingers. Compared with wrist and forearm movements, finger movements give rise to smaller signal amplitude. Therefore, in the current study, the sampling frequency will be investigated through analyzing the finger movement classification accuracy. Moreover, FMG signals for hand motion classification tend to use 16 or 32 channels. However, it is still not clear how the channel number would influence the accuracy, which becomes another point for the current study.

Pattern recognition technology is usually used to predict hand gestures from FMG signals. As summarized in Table 1, RMS is one of the most commonly used FMG feature [11,13,16], and SVM [9,10,13,16,17,21] and LDA [13,14,15,16,17,18,19,20,22] are the most commonly used classifiers for FMG based hand gesture classification. Although different features (or systems without feature extraction) and different classifiers are applied in this field, one hardly finds which type of feature or classifier outperforms the others as seen in Table 1.

## 2. Materials and Methods

In this paper, a 16-channel FMG signal acquisition system is developed to acquire FMG signals of gestural movements.

### 2.1. Sensing Unit

The sensing unit is a sophisticated component that converts the pressure signal caused by muscle contraction into electrical signal. The force sensing resistor (FSR, Interlink Electronics, Inc, Camarillo, CA, US) that is made of robust polymer film are utilized in this study, which converts the change of pressure into specific resistance change. This series of sensors provide a relatively large sensitivity range (0.2–20 N), the sensing signal grows logarithmically with increasing pressure. However, it loses repeatability at high force range due to unavoidable hysteresis.

However, for smaller forces (0–10 N), their transfer function is almost linear. Besides, it has properties of low drift, softness and low cost, which makes it very suitable for FMG signal capturing, as can been seen in Table 2. During all the models of FSR sensor, FSR 400 has smaller contact area, and therefore it can be placed on the skin surface in a high-density arrangement. It is the reason why FSR 400 is selected in this study for FMG signal sensing.

### 2.2. System Architecture

**Figure 1:**
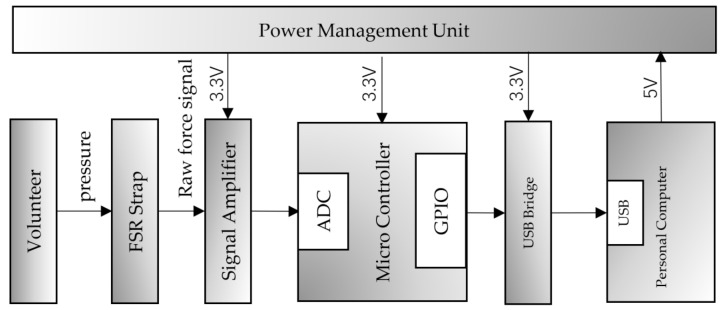
The hardware architecture for FMG acquisition.

**Figure 2:**
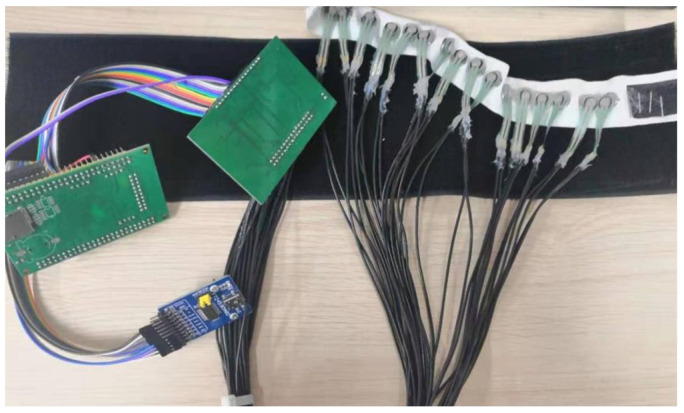
The hardware prototype for FMG acquisition.

A FMG signal acquisition prototype is constructed in this study for FMG signal acquisition. The internal structure of the system is shown in Figure 1. The system consists of an FMG cuff, a signal amplification circuit, a STM32 microcontroller, a USB transmission module UMFT 245 (Future Technology Devices International Ltd., Glasgow, UK) and a personal computer. As shown in Figure 2, the FMG cuff is a flexible belt embedded with 16 sensors (i.e., FSR 400). The flexibility of the belt makes it suitable for a large range of arm circumferences. Meanwhile, this kind of arrangement also guarantees that all sensors can be placed in a relative stable position corresponding to the muscles. Signal amplification and filtering circuits are designed for FMG signal conditioning, where a 22 kΩ resistor and an FSR sensor make up a voltage dividing circuit [12]. The A/D converters (12 bits resolution) embedded in a STM32 micro-controller are used for signal digitalization, and then all signals are transmitted to personal computer by USB port via a cable, for which a UMFT245 based USB bridge is taken for signal transmission.

**Figure 3:**
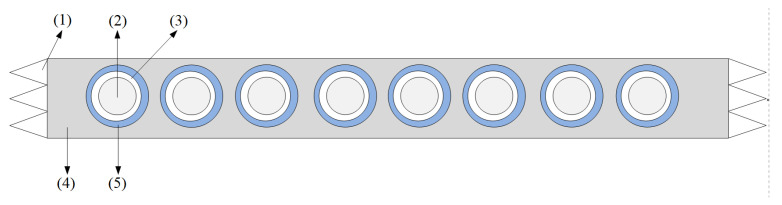
The sensing armband structure, including (**1**) Velcro, (**2**) hemispherical silicone pad, (**3**) force morphology sensor, (**4**) elastic fabric band and (**5**) flat button. The mechanical coupler is placed on the sensing head.

The movement of the fingers gives rise to the contraction of the forearm muscles, which causes the variation of the pressure between skin surface and the sensing unit. However, the contact surface of the original FSR sensor is flat, which negatively influences FMG signal quality. Therefore, in this paper, a flexible mechanical coupler [2,31] is added on the FSR sensor to enhance the contact quality, as shown in Figure 3.

The mechanical coupler made of silicone is molded into a hemisphere with a diameter of 0.7 cm. The flat area of the coupler is placed in the sensing area of the FSR, while the protruding part is used to contact with the skin. The hemispherical structure makes the sensor more sensitive due to higher contact pressure and more uniform pressure distribution on the sensing area.

### 2.3. Experimental Setup

**Figure 4:**
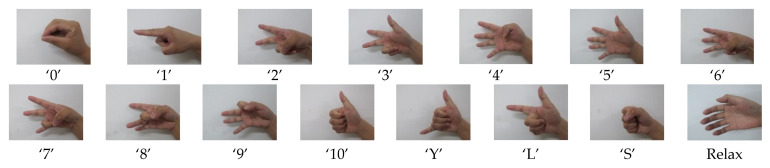
The hand gestures for data capturing.

As shown in Figure 4, fifteen hand gestures labeled by a number (0 to 10), letter (Y, L and S) and relaxed state that would be commonly used during daily life are put forward in [5,32].

**Figure 5:**
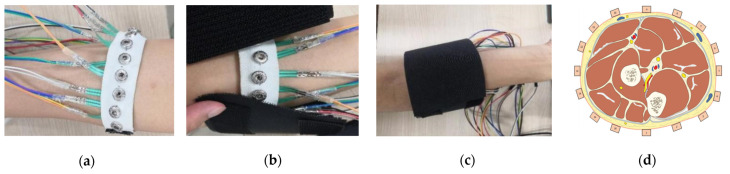
The wearing of FMG band on the forearm: (**a**) wearing the sensing armband; (**b**) covering the sensing armband by a non-stretchable fabric; (**c**) covered sensing armband; and (**d**) the positioning order of 16 FSR sensors.

Ten subjects volunteered for the experience, and they are all right-handed. Before data recording, the research assistant explains the purpose of the experiment to participants, and then obtains written consent. With the help of the research assistant, participants wear the sensing armband (Figure 5a), which matches the maximum arm circumference of the right arm. The positions of each FSR sensor corresponding to the forearm muscle are demonstrated in Figure 5d. A non-stretchable fabric covers the sensing armband to enhance the contact quality (Figure 5b,c). After wearing the sensing armband, subjects sit on a chair and put the elbow on a table to implement gestures. During the whole stage of signal recording, subjects are asked to keep stable as far as possible to alleviate the negative effect of artifacts.

During data recording, each gesture is maintained for five seconds and followed by a rest period of five seconds, and each gesture repeats 10 times. To avoid muscles fatigue and to perform gestures in a natural manner, the participants are allowed to rest for two minutes between two gestures. Additionally, participants are required to change the rest state to action state within one second.

### 2.4. Data Analysis and Processing

**Figure 6:**
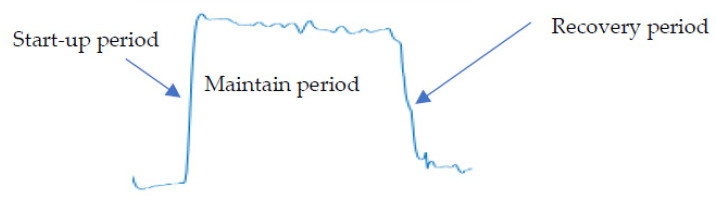
The change of FMG signal for one repetition of a gesture. It contains start-up period, maintain period, and recovery period.

A complete cycle of the gesture FMG signal includes three parts: start-up period, maintain period and recovery period, as shown in Figure 6. The study focuses on static actions, so only the signal during the maintain period is segmented for hand motion classification. The main reason is that the signal during start-up period and recovery period has large variation, which would decrease the overall classification accuracy [33]. Besides, the correct outputs during static period can quickly overwhelm incorrect ones during these transient stages, because there are very limited samples during transient stages.

In this paper, the original FMG signal is downsampled in both the temporal domain and spatial domain. The investigated sampling frequencies are 1 kHz, 0.5 kHz, 0.1 kHz, 10 to 90 Hz stepped by 10 Hz and 1 to 9 Hz stepped by 1 Hz. Before downsampling, 1 kHz original signals are processed by 4th order Butterworth filters, and the cutoff frequency is double of the targeted downsampling frequency. Moreover, the number of channels after spatial downsampling is 16, 8, 4 and 2, respectively.

After downsampling, the RMS [10,11,16] feature is extracted from the signal using sliding window technology. As an alternative, the MEAN feature is also adopted in this study, which is the same as the mean absolute value (MAV) feature of EMG signal analysis [34,35,36,37], because all the FMG values are positive. The equations for feature extraction are as follows.

(1) $$RMS=\sqrt{\frac{1}{N}\sum_{i=1}^{N}x_{i}^{2}},\;$$

(2) $$MEAN=\frac{1}{N}\sum_{i=1}^{N}x_{i},$$

where $x_{i}$ is the sample value, and *N* is the length of the time window, which also represents the total number of samples within a window. When the sampling frequency is within the range of 10 Hz~1 kHz, the length and increment are exactly set to 500 ms and 20 ms, respectively. When the frequency ranges from 1 Hz to 9 Hz, the window length and increment are set close to 0.5 s and 0.2 s, because there may be not enough sampling points within 500 ms.

Five classifiers, including Support Vector Machine (SVM), K-Nearest Neighbors (KNN), Random Forest (RF), Linear Discriminate Analysis (LDA) and Naive Bayesian (NB) are employed to evaluate the effect of downsampling on gesture recognition. To obtain more accurate recognition, ten-fold cross-validation is used in this paper specifically. A ten-fold cross-validation implementing process is applied for the experiment. The number of folds is consistent with the number of repetitions of each gesture during data collection.

## 3. Results

### 3.1. The Impact of Sampling Frequency on Classification Accuracy

**Figure 7:**
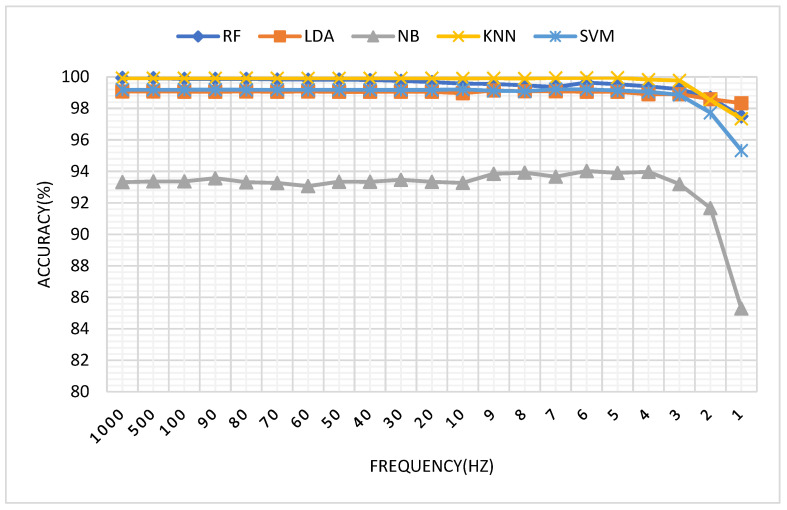
The tendency of classification accuracy (%) along with the decreasing of sampling frequency.

Figure 7 shows the tendency of classification accuracy along with the decrease of sampling frequency. As can be seen from Figure 7, RF, NB, KNN and SVM demonstrate a similar tendency. The accuracy keeps stable for frequencies above 5 Hz, and below 5 Hz it starts decreasing. Among these classifiers, LDA demonstrates a relative stable performance, and no clear accuracy decrease can be found even when the sampling accuracy is lower than 2 Hz. It is possibly because that LDA is not sensitive to the number of samples. Moreover, in the comparison with other classifiers, NB obtains the lowest classification accuracy (about 93% before 5 Hz), which is possibly because that small deviation of the mean and variance between training data and testing data would severely influence the prediction performance of a trained Bayesian model. It is also found that KNN and RF obtain almost the same accuracy curve, and their accuracy is higher than that of SVM by about 1%, which is possibly because SVM is basically a type of 2-class classifier, and additional algorithms are required for classifying multiple classes. In the rest of the paper, KNN and SVM are used as the representatives for classification.

### 3.2. The Impact of Channel Number on Classification Accuracy

This study investigates how the number of channels influences the classification accuracy, as reported in Table 3. This experiment guarantees that all selected channels are evenly distributed around the forearm.

As seen from the table, the decreasing of number of channels has a large influence on the accuracy. The accuracy reduces by 6.92% and 20.85% for KNN and SVM in the comparison between 16 and 2 channels at 1 kHz frequency. It can be seen that SVM is more sensitive to the number of channels than KNN. When the number of sampling channels is 4, with the frequency declines from 1 kHz to 5 HZ, the accuracy drops by 1.01% and 0.31% for KNN and SVM, respectively. This result indicates that the number of channels would influence the accuracy more significantly than the sampling frequency.

### 3.3. The Impact of Channel Combination on Classification Accuracy

**Figure 8:**
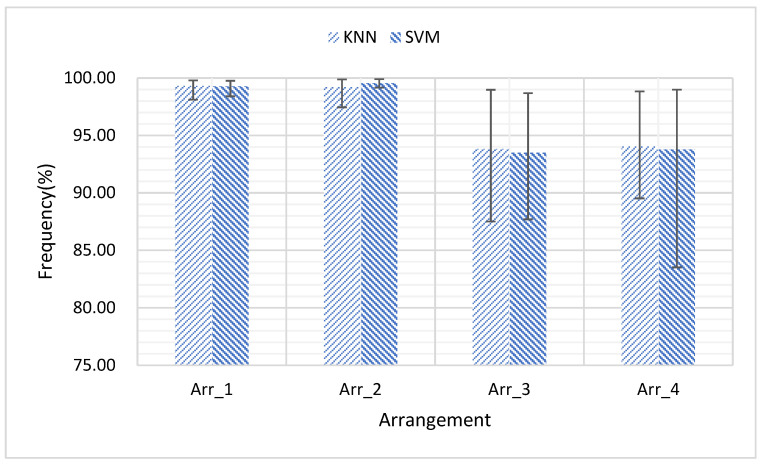
When selecting four channels in four types of arrangement, the classification accuracy is compared. Arr_1 indicates the selection of channel 1, channel 5, channel 9 and channel 13. Arr_2 indicates the selection of channel 3, channel 7, channel 11 and channel 15. Arr_3 indicates the selection of channel 2, channel 6, channel 10 and channel 14. Arr_4 indicates the selection of channel 4, channel 8, channel 12 and channel 16. The numbering of the channels is shown in Figure 5d.

This study also investigates if the accuracy can be improved through optimizes the selected channels. Four channels’ arrangements (Arr_1, Arr_2, Arr_3, and Arr_4) are compared, as seen in Figure 8. It can be seen that Arr_1 and Arr_2 significantly outperform Arr_3 and Arr_4. However, there is no obvious difference between Arr_1 and Arr_2, or between Arr_3 and Arr_4.

### 3.4. Individual Differences for FMG Based Hand Gesture Classification

**Figure 9:**
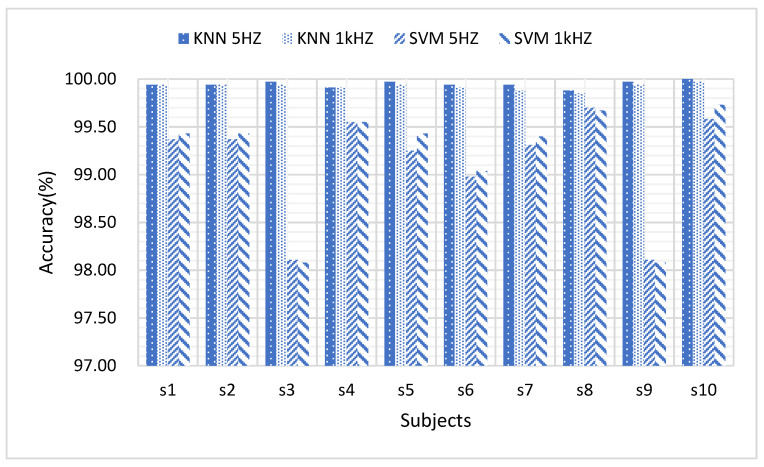
Comparison of classification accuracy of different subjects by KNN and SVM classifiers at sampling frequency of 1 kHz and 5 Hz.

As can be seen from Figure 9, it seems that KNN is more robust than SVM with respect to the variance among subjects, which is possibly because SVM is basically a type of 2-class classifier. It is also found that the sampling frequency (1 kHz vs. 5 Hz) would not influence the accuracy very much, in the comparison with subject variance and classifier variance.

## 4. Conclusions

Most researchers set the sampling frequency to 100 Hz when acquiring FMG signals. Carlo et al. [26] has pointed out that the minimum sampling frequencies of FMG signals for the study of isometric actions are about 55 Hz. In the current study, the sampling frequency are down sampled from 1 kHz to 1 Hz to investigate how the sampling frequency influence the classification of static finger movements and find that 5 Hz sampling frequency can be enough. Besides, this study demonstrates that increasing the number of FMG channels can be a good choice to improve the hand gesture classification performance, and eight channels is suggested when system complexity and accuracy are both considered. Further, the optimization of FMG sensor placement can significantly improve the accuracy. More than 5% accuracy difference can be seen with different sensor placement with four sensors.

## Figures and Tables

**Table 1:** Sampling rates and number of sampling channels in the literature.

Studies	Sampling Frequency (Hz)	Number of Channels	Number of Gestures	Features	Classifiers	Classification Accuracy (%)
Li, 2012 [9]	100	32	17	-	SVM	99
Ha, 2018 [10]	25	-	-	-	SVM	95
Carlo, 2018 [11]	50	10	6	-	LDA, SVM, TB ^4^ and NN ^5^	87.7
Carlo, 2014 [12]	1000	8	6	-	ELM ^11^	92.33
Carlo, 2017 [13]	1000	8	45	RMS ^1^	LDA ^2^ and SVM ^3^	84.3
Radmand, 2016 [14]	20	126	8	-	LDA	99.7
Jiang, 2018 [15]	15	16	16	-	LDA	82
Carlo, 2018 [16]	15	18	15	RMSE ^6^ and R ^2–7^	LDA, SVM and RF ^8^	90
Ahmadizadeh, 2017 [17]	10	16	10	-	KNN ^9^, SVM and LDA	81.1
Ghataurah, 2017 [18]	10	16 × 5	11	-	LDA	-
Chengani, 2016 [19]	10	16	8	-	LDA	95
Mena, 2017 [20]	10	16	7	RMS	-	94
Anvaripour, 2018 [21]	10	8	6	PSD ^10^	SVM	93
Ahmadizadeh, 2019 [22]	10	16	6	-	LDA	79.2
Kant, 2019 [23]	100	8	8	VR ^12^	-	-
Cho, 2016 [24]	10	88	11	-	LDA	89

^1^ is Root Mean Square; ^2^ is Linear Discriminant Analysis; ^3^ is Support Vector Machine; ^4^ is bagging of decision trees (TreeBagger); ^5^ is Neural Network; ^6^ is Root Mean Square Error; ^7^ is two output of statistic test; ^8^ is Random Forest; ^9^ is K-Nearest Neighbor; ^10^ is Power Spectral Density; ^11^ is Extreme Learning Machine; ^12^ is Variance Ratio.

**Table 2:** The selection of FSR sensors for FMG acquisition.

Sensor Model	Studies
FSR 400	[9,27,28]
FSR 400 short	[4,23,28,29]
FSR 402	[2,11,12,30]

**Table 3:** Hand gesture classification accuracy by KNN and SVM under different combination of channel number and sampling frequency, where 8_KNN as an example indicates using 8 channels and KNN classifier.

Accuracy (%)	1 kHz	100 Hz	10 Hz	5 Hz	3 Hz	2 Hz	1 Hz
16_KNN	99.92	99.91	99.90	99.95	99.77	99.63	99.33
16_SVM	99.18	99.18	99.20	99.13	98.86	98.54	97.41
8_KNN	99.85	99.81	99.49	99.42	99.01	98.50	97.16
8_SVM	98.13	98.71	98.49	98.18	93.11	96.01	93.11
4_KNN	99.33	99.28	99.95	98.93	98.32	97.58	95.73
4_SVM	93.78	93.76	93.50	93.47	86.32	90.29	86.32
2_KNN	93.00	92.03	91.94	92.02	90.33	88.77	85.97
2_SVM	78.33	78.30	77.97	77.92	70.20	74.65	70.20
